# In vivo measurement of stent length by using intravascular ultrasound

**DOI:** 10.1186/s43044-019-0036-9

**Published:** 2019-12-19

**Authors:** Magdy Algowhary, Salma Taha, Hosam Hasan-Ali, Akihiko Matsumura

**Affiliations:** 1 Department of Cardiovascular Medicine, Faculty of Medicine, Assiut University, Asyut, 71516 Egypt; 2 Department of Cardiology, Kameda Medical Center, Higashi-cho 929, Kamogawa, Chiba 296-8602 Japan

**Keywords:** IVUS, Stent length, Stent shortening

## Abstract

**Background:**

What happens to stent length when deployed in a coronary artery? It is the aim of this study.

**Results:**

Consecutive 95 balloon-expandable stents (BES) were studied by intravascular ultrasound (IVUS) imaging. The stent length was measured from the longitudinal view in two ways: (1) edge-to-edge length (E-E) measured between distal and proximal stent frames located at one IVUS quadrant and (2) area-to-area length (A-A) measured between distal and proximal stent frames located at two or more IVUS quadrants. IVUS measurements were compared with the manufacturer-stated length (M-L). The median E-E length was significantly longer than M-L, 18.76 mm [interquartile range (IQR) 15.65–23.60] versus 18.00 mm (IQR 15.00–23.00), respectively, *p* < 0.0001. Also, the median A-A length was significantly longer, 18.36 mm (IQR 15.19–23.47), *p* < 0.0001, than M-L. Moreover, the E-E length was significantly different from A-A length, *p* < 0.0001. Among the stent groups, the differences were significantly present in all drug-eluting stent and bare metal stent (BMS) comparisons, *p* < 0.0001, except the A-A length versus M-L in BMS only. By multivariate analysis, the predictors of difference in stent length were as follows: lesion length, *p* = 0.01; pre-intervention minimal diameter of the external elastic membrane (EEM), *p* = 0.03; lesions present in the left anterior descending branch, *p* = 0.03; and M-L, *p* = 0.04.

**Conclusions:**

In the present study, the length of BES measured by IVUS was significantly different from the manufacturer-stated length. In addition to the manufacturer length, other important factors such as lesion length, pre-intervention diameter of EEM, and affected vessel determine the stent length.

## Background

Coronary stenting is an important tool in the management of coronary artery disease. Stent geometry plays an important role in treating different lesions. The most recent guidelines stress on the importance of usage of intravascular examination (IVUS) on the evaluation of both lesion and stent lengths [1]. The researchers studied the transverse stent dimension, stent diameter, and found that the minimal stent diameter measured by IVUS was significantly smaller than that predicted by the in vitro compliance charts, and these differences were independent of stent manufacturer, deployment pressure, and stent length [2]. The longitudinal stent dimension is a subject for further studies. Cases with longitudinal stent compression and elongation were detected and were described as longitudinal stent deformation (LSD). The LSD has important clinical implications and should be treated accordingly [3–6]. In vitro studies using 3D computational fluid dynamics modeling indicated that foreshortening induced by the orientation of stent struts with respect to the direction of blood flow is associated with an increase in the area of the vessel subjected to low wall shear stress and high wall shear stress gradient, factors that have been correlated with subsequent neointimal hyperplasia. It was found that the angle between axially aligned stent struts and the main direction of the blood flow varied with the deployed stent length. Compared with their ideal computational stent architecture, the known stent length, the foreshortened stents demonstrated that the stent struts were progressively misaligned with the direction of blood flow increasing the deleterious distribution of wall shear stress and wall shear stress gradient [7], and these areas predicted subsequent in vivo neointimal proliferation [8]. Nevertheless, longitudinal stent foreshortening is evident in self-expandable stents keeping in mind that the whole lesion should be covered. However, in balloon-expandable stents (BES), longitudinal stent foreshortening needs further assessment.

The aim of this study is to measure the length of BES after deployment using IVUS imaging and compare it with the manufacturer-stated length (M-L) in order to determine whether the length after deployment is identical to, shorter than or longer than the M-L.

## Methods

### Study population

Patients with stable and unstable angina pectoris and myocardial infarction due to native coronary artery disease were included in this study. All consecutive patients who underwent IVUS-guided percutaneous coronary intervention were included in this study. The study was conducted after a written informed consent was taken from every patient who underwent IVUS-guided percutaneous coronary intervention. Lesions treated by a single bare metal stent (BMS) or drug-eluting stent (DES) were included. Two- and three-vessel disease was included as long as one stent per lesion was done. The following cases were excluded: lesions treated by two overlapped stents, poor IVUS image quality, manual pullback, presence of heavy calcification at the stent edges, study with non-uniform rotational distortion (NURD), and IVUS study with non-uniform or interrupted pullback. The ethics committee of the institutional review board of our hospital approved this research.

### Coronary stent procedures

The cases were selected from real-world cases. On the basis of diagnostic coronary angiography, patients underwent percutaneous coronary intervention if one or more major coronary arteries had stenosis of at least 70% and were suitable for revascularization. Heparin was administered intravenously to maintain an activated clotting time more than 300 s. BES was deployed directly or after predilatation. No exclusion was made for stent type, strut thickness, radial force, or number of strut connectors. Coronary stenting was guided by IVUS examination before and after stenting. If incomplete stent expansion/apposition detected by IVUS or more than 10% residual angiographic stenosis was seen inside the stented vessel, further balloon dilatation was done to achieve optimal stenting results.

### Quantitative coronary angiographic analysis

Prestenting and poststenting cineangiograms were analyzed using a quantitative coronary angiographic automated edge detection algorithm (QCACMSR, version 4, MEDIS, Leiden, The Netherlands). The outer diameter of the contrast-filled catheter was used for calibration. Reference diameter, minimal lumen diameter, and before and after intervention percentage diameter stenosis were measured from multiple projections, and the results from the narrowest view were recorded.

### IVUS procedure and analysis

The examination was performed using a 2.5-F IVUS catheter operating on a frequency of 40 MHz. After the administration of intracoronary nitrates, the transducer was positioned in the distal vessel, at least 10 mm distal to the stent, and withdrawn at a rate of 0.5 mm/s with the use of a motor drive (CardioVascular Imaging System ClearView Ultra, CVIS, Boston Scientific, Fremont, CA, USA) to the aorto-ostial junction. On a computer screen, manual planimetry was performed to measure the external elastic membrane (EEM) and lumen areas in all frames. The computer program calculated the measurements for each frame and saved them. Stent length was measured by two methods. In the first method, from the short-axis view, the first distal frame with the first stent struts located at one quadrant was identified and marked at the long-axis view. Then, the proximal frame with the last stent struts located at one quadrant was identified and marked at the long-axis view too. The distance between the two frames was measured at the long-axis view and was termed edge-to-edge (E-E) length. In the second method, the length was measured between the distal and the proximal frames with the first and the last stent struts seen at more than one quadrant in the short-axis view and was termed area-to-area (A-A) length. If the first and/or the last stent struts were seen only at more than one quadrant, the frame was used for both distances, E-E and A-A. The same method was used before by Dvir et al. [9]

### Assessment of reproducibility

Reproducibility and intraobserver variability of IVUS measurements were tested at ten successive frames (1-mm segment) randomly selected from every patient IVUS study. The same person performed a repeated analysis at 4 months apart. The differences in the measurements were as follows: mean lumen area (0.04 ± 0.94 mm^2^), mean EEM area (0.23 ± 0.91 mm^2^), and mean plaque-media area (0.18 ± 0.98 mm^2^). The intraclass correlation coefficient for repeated measurement of the lumen area was 0.97, of the EEM area was 0.99, and of the plaque-media area was 0.98.

### Statistical analysis

Categorical variables are presented as frequencies, and continuous variables are reported as median (interquartile range, IQR) and mean ± SD (for normally distributed variables). For the comparisons of continuous variables, Wilcoxon signed-ranks test with exact method and a two-tailed paired-samples *t* test were used. Correlations between variables are described with the use of Spearman and Pearson correlations. Variables with *p* < 0.1 were used in the general linear model to obtain the predictors of the differences between the stent length measured by IVUS, E-E length, and the M-L. All statistical tests were two-sided, and *p* value < 0.05 was considered significant. All analyses were performed using SPSS package 22 (SPSS Inc., Chicago, IL, USA).

## Results

**Fig. 1:**
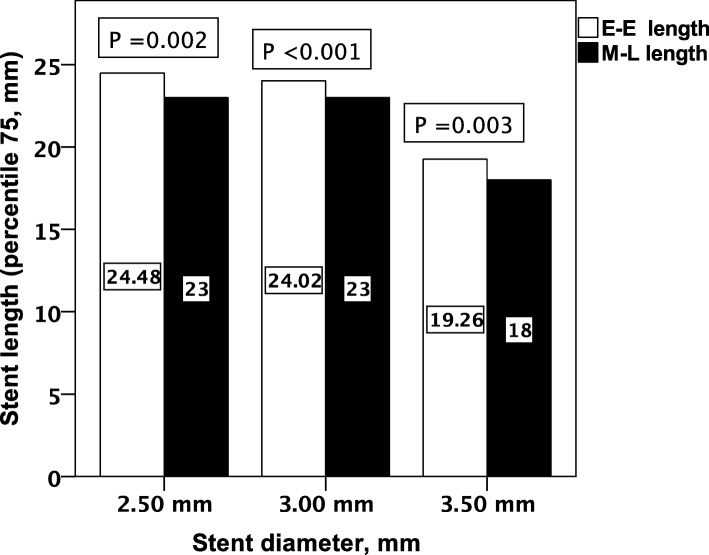
Comparison between IVUS-measured stent length and manufacturer stent length according to the stent diameter. E-E length = edge-to-edge stent length by IVUS, M-L length = manufacturer-stated length

The included patients were 130 consecutive patients; however, 40 patients were excluded from the study. Excluded patients were due to the presence of calcification interfering with proper identification of stent edge in 20 patients, interference of stent imaging by the guiding catheter in 16 patients with ostial located stents, use of cutting balloon in 2 patients, and interrupted IVUS pullback in 2 patients. The study included 90 patients who underwent 95 stent implantations. Their ages ranged from 43 to 84 years; their baseline data are presented in Table 1. Procedural and IVUS data are summarized in Tables 2 and 3. The stent diameters vary from 2.5 to 4.0 mm, and the stent lengths range from 8.0 to 30.0 mm. Both IVUS measurements of stent lengths, E-E and A-A, are significantly longer than the manufacturer-stated length (M-L) and the calculated shortened length (derived from the manufacturer stent shortening data) as in Table 4. The IVUS measurements were significantly longer than the manufacturer measurements in both DES and BMS patients except the A-A length versus the M-L in BMS patients (*p* = 0.4). Figure 1 shows the comparisons of the E-E length versus the M-L according to the stent diameters.

**Table 1:** Clinical data

		Patients (*n* = 90)
Age (years)		66.6 ± 10.0
Sex (%)	Men	74 (82.2)
	Women	16 (17.8)
Smokers (%)		51 (56.7)
Hypertension (%)		60 (66.7)
Diabetes mellitus (%)		32 (35.6)
Dyslipidemia (%)		61 (67.8)
Family history (%)		14 (15.6)
Chronic stable angina pectoris (%)		39 (43.3)
Unstable angina pectoris (%)		29 (32.2)
Acute myocardial infarction (%)		22 (24.4)
Ejection fraction		0.62 ± 0.12
Statin (%)		52 (57.8)
β-Blocker (%)		27 (30)
Calcium antagonist (%)		39 (43.3)
ACE inhibitor (%)		21 (23.3)
ARB (%)		32 (35.5)
Cholesterol, mg/dl		190.06 ± 35.94
LDL cholesterol, mg/dl		120.28 ± 28.34
HDL cholesterol, mg/dl		48.44 ± 13.34

Data are given as mean ± SD or percentage

*ACE* angiotensin-converting enzyme, *ARB* angiotensin receptor blocker, *β-blocker* beta-blocker, *HDL cholesterol* high-density lipoprotein cholesterol, *LDL cholesterol* low-density lipoprotein cholesterol

**Table 2:** Stenting data

		Lesions (*n* = 95)
Lesion site (%)	LAD	55 (57.9)
	LCX	18 (18.9)
	RCA	22 (23.2)
Lesion type^a^ (%)	Low risk	41 (43.2)
	Moderate risk	46 (48.4)
	High risk	8 (8.4)
Lesion length, mm		9.09 (7.0–13.25)
Reference diameter, mm		3.13 ± 0.64
Prestent MLD, mm		0.82 ± 0.32
Prestent diameter stenosis (%)		63.06 ± 14.87
Poststent MLD, mm		2.56 ± 0.44^†^
Poststent diameter stenosis (%)		10.49 ± 5.50^†^
Drug-eluting stent (%)		38 (40)
Bare metal stent (%)		57 (60)
Stent diameter, mm (%)	2.5 mm	14 (14.74)
	3.0 mm	54 (56.84)
	3.5 mm	23 (24.21)
	4.0 mm	4 (4.21)
Stent length, mm (%)	8.0 mm	2 (2.1)
	9.0 mm	2 (2.1)
	12.0 mm	5 (5.3)
	13.0 mm	4 (4.2)
	15.0 mm	18 (18.9)
	18.0 mm	32 (33.7)
	20.0 mm	3 (3.2)
	23.0 mm	18 (18.9)
	24.0 mm	3 (3.2)
	25.0 mm	1 (1.1)
	28.0 mm	6 (6.3)
	30.0 mm	1 (1.1)
Stent strut (wire) thickness, mm		0.14 (13.0–16.0)
Stent deployment pressure, atm		16.0 (14.0–18.0)
Poststent balloon dilatation (%)		19 (20%)

Data are given as mean ± SD, percentage, or median (IQR)

*LAD* left anterior descending artery, *LCX* left circumflex artery, *MLD* minimal lumen diameter, *RCA* right coronary artery

^a^American College of Cardiology/American Heart Association (ACC/AHA) classification [10]

^†^*p* < 0.0001 (compared with the prestent measurements)

**Table 3:** IVUS data

Lesion segment	Prestenting	Poststenting
Calcified plaque-media (%)	41 (43.16)	
Lumen area, mm^2^	4.33 ± 2.00	7.91 ± 2.48*
Minimal lumen diameter, mm	2.05 ± 0.47	2.91 ± 0.46*
Maximal lumen diameter, mm	2.56 ± 0.57	3.45 ± 0.52*
EEM area, mm^2^	14.06 ± 4.37	16.84 ± 4.61*
Minimal EEM diameter, mm	3.94 ± 0.17	4.32 ± 0.58*
Maximal EEM diameter, mm	4.52 ± 0.66	4.94 ± 0.68*
Plaque-media area, mm^2^	9.74 ± 3.76	8.93 ± 3.21^†^
Proximal reference segment		
Calcified plaque-media (%)	32 (33.68)	
Lumen area, mm^2^	9.03 ± 3.29	8.77 ± 2.99
EEM area, mm^2^	17.29 ± 5.05	17.82 ± 5.01^†^
Plaque-media area, mm^2^	8.26 ± 3.37	9.05 ± 3.56*
Distal reference segment		
Calcified plaque-media (%)	41 (43.16)	
Lumen area, mm^2^	6.30 ± 2.07	6.91 ± 2.13*
EEM area, mm^2^	11.89 ± 3.84	12.78 ± 3.90*
Plaque-media area, mm^2^	5.59 ± 2.93	5.87 ± 2.69

Data are given as mean ± SD or percentage

*EEM* external elastic membrane

*p < 0.001 (compared with the prestent measurements);

^†^*p* < 0.01 (compared with the prestent measurements)

**Table 4:** IVUS stent length measurements and manufacturer stent length (manufacturer-stated length and foreshortened length)

	Measurements, mm
E-E length*	18.76 (15.65–23.60)
A-A length^†^	18.36 (15.19–23.47)
M-L length	18.0 (15.0–23.0)
Calculated foreshortened stent length	17.84 (14.59–22.79)

Data are given as median (IQR)

*E-E length* edge-to-edge stent length, *A-A length* area-to-area stent length, *M-L length* manufacturer-stated length

*p < 0.0001 is for E-E length versus M-L length, calculated foreshortened stent length and A-A length

^†^*p* < 0.0001 is for A-A length versus M-L length and calculated foreshortened stent length

From clinical, laboratory, stenting, and IVUS data, the following statistically important variables were used to identify the predictors of the difference in stent length between the E-E and M-L: gender (*p* = 0.06), smoking (*p* = 0.007), statin therapy (*p* = 0.04), stented vessel (*p* = 0.07), stent type (*p* = 0.009), stent diameter (*p* = 0.004), stent strut thickness (*p* = 0.003), M-L (*p* = 0.06), deployment pressure (*p* = 0.04), lesion length (*p* = 0.03), lesion plaque type by IVUS (*p* = 0.05), prestenting lesion mean lumen area (*p* = 0.03), mean minimal lumen diameter (*p* = 0.01), mean maximal lumen diameter (*p* = 0.04), mean EEM area (*p* = 0.003), mean minimal EEM diameter (*p* = 0.003), mean maximal EEM diameter (*p* = 0.0005), mean plaque-media area (*p* = 0.02), mean distal reference EEM area (*p* = 0.007), and mean distal reference plaque-media area (*p* = 0.01). The significant independent predictors by multivariate analysis were lesion length, prestenting lesion mean minimal EEM diameter, lesions present in the left anterior descending vessel, and M-L (Table 5).

**Table 5:** Predictors of difference in stent length

	*p* value
Lesion length	0.01
Prestenting minimal EEM diameter	0.03
Lesions present in the LAD vessel	0.03
Manufacturer-stated length (M-L)	0.04

*EEM* external elastic membrane, *LAD* left anterior descending vessel

## Discussion

The principal finding of this study is that, after deployment of BES in coronary arteries of real-world patients, the stent length measured by IVUS is significantly different from the M-L. Both measurements of stent length represented by the longitudinal distance between the proximal and the distal stent edges (E-E length) and stent borders (A-A length) are significantly longer than the manufacturer-stated length. The reason for this may be the elongation of the stent during high-pressure balloon inflation. It was seen after stent deployment under high pressure, and consequently, stent elongation would occur [11]. Of note, 88 stents (92.6%) were deployed under high pressure (14 atm or more) in our trial. In addition to manufacturer-stated length, other factors were important in determining longer stent lengths such as lesion length, deployment of the stent in the left anterior descending vessel, and vessel size. More recently, the interaction between the stent and the vessel wall has been illustrated as a complex interaction that presumably leads to stent elongation. The stent length has been determined by the plaque composition and eccentricity at the lesion site [12]. In contrary to BES, serial IVUS examination of self-expandable stents revealed a significant stent shortening during long-term follow-up [13], which might need stent oversizing at the time of implantation [14].

There may be a concern regarding the withdrawal speed of the IVUS catheter during examination especially in long lesion, tortuous vessel, small and diffusely diseased vessel. These factors may interfere with the automatic pullback speed rendering it more slowly, and consequently, the stent length will be longer. However, in our study, the median lesion length was short, 9.09 mm, most of the lesion types were not complex [types A and B; 87 lesions (91.6%)]; the mean reference vessel was not small, 3.13 mm; the smallest stent diameter, 2.5 mm, was used in only 14 lesions (14.74%); and the long stents, ≥ 20 mm, were used in 32 lesions (33.68%); thus, all these factors made a smooth non-interrupted IVUS catheter withdrawal. Of note, the stent length was longer in 79 stents (83.2%) of the cases. Moreover, another concern regarding the longitudinal movement of the IVUS catheter with each heartbeat is IVUS catheter positions which may change during cardiac cycle. Theoretically, the problem of longitudinal movement can be minimized with EKG gating and measurements made only at end-diastole, however, Kaple et al. [15] found similar results on measuring stent length by using ECG-gated IVUS with standard greyscale IVUS. Also, it was not superior to the standard IVUS.

The axial resolution of IVUS frame is important to identify stent struts and hence stent length in this study. The axial and lateral resolutions depend on the frequency of the ultrasound beam. In our study, the frequency of the IVUS catheter is 40 MHz which gives a satisfactory stent imaging. The commercially available IVUS catheters use 20–40 MHz providing 70–200 μm and 200–400 μm lateral resolution and 5–10 mm imaging depth. Optical coherence tomography (OCT) uses back-scattered infrared light to generate high spatial resolution, 10–30 μm, but shallow penetration depth. Ultra-high frequency IVUS at 80 MHz gives higher axial resolution thus improving stent visualization and a comparable penetration depth to OCT, 2 mm. New IVUS catheter design, multi-frequency IVUS, can use both low and high frequencies (35/90 MHz, 35/120 MHz, and 35/150 MHz) in order to get both advantages of high penetration and high axial resolution [16].

The longitudinal stent architecture is not always straight as in vitro analysis, but it may be curved and even angulated in either end-to-end or side-to-side directions depending on the affected vessel, the lesion site, and the lesion characters. Also, the position of the IVUS catheter and the ultrasound beam may not be perpendicular to the short axis of the stent struts, i.e., the IVUS catheter may not pass through the center or may change with the cardiac cycle. To overcome these problems, the stent length was measured in two ways. The start point and the end point were identified at two levels: (1) the start/end of viewing of stent strut, i.e., the E-E frame, where any strut could be seen at even one quadrant was considered the start/end of E-E length; (2) the frame where stent struts could be seen at two or more IVUS quadrants, i.e., A-A frame, was considered the start/end of A-A length. Thus, not only one length was used to measure stent length but two measurements. The stent edges would lie definitely at either E-E frame or A-A frame. Interestingly, both E-E stent length and A-A stent length were longer than the manufacturer stent length, M-L length. Moreover, the same method was used before in Dvir’s trial in 2014 [9]. Of note, the E-E length was significantly longer than the A-A length; consequently, the distance between the A-A length and the E-E length added a significant length to the stent. Although that distance might have a concern regarding the angel between the IVUS catheter and the stent struts (i.e., the ultrasound beam was not perpendicular to the stent), it was functionally covered part of the vessel and should not be left. Nevertheless, the A-A length was significantly longer than both manufacturer-stated lengths, the label stent length and the calculated foreshortened stent length too. Thus, whatever the method used to measure the stent length, it was significantly longer than the manufacturer-stated length data. Tanaka et al. [17] measured the stent length by using a similar pullback device (CVIS) in 45 patients. In contrary to our findings, the length was significantly shorter. However, the stent length was measured between the first complete circumferential appearance of the stent struts and the disappearance of complete circumferential visualization of the stent struts. The complete circumferential appearance of stent struts means the struts should be seen in the four quadrants of IVUS image while the measurements of our study were between the stent struts seen in one quadrant (E-E length) and in two or more quadrants (A-A length), i.e., not arbitrary seen in the four quadrants. We frequently found the complete circumferential stent struts lied proximal and distal to the first and the last stent struts at the distal and the proximal stent edges, respectively. This made the measurements shorter because it excluded part of the stent seen in two and three IVUS quadrants at both edges. Moreover, we do not know exactly the shape of the circumference of the stent at both stent edges and whether or not it is perpendicular to the vessel wall. Also, we do not know the plane of the ultrasound beam in relation to both the vessel wall and the stent. Besides, the patients’ number in our study was much more than the previous study, 90 patients versus 45 patients. Nevertheless, the correlations between the known M-L and the stent lengths measured in this study were *r =* 0.89 for the E-E length (*p* < 0.001) and *r* = 0.91 (*p* < 0.001) for the A-A length. Tanaka et al. obtained nearly a similar correlation, *r* = 0.92.

Recently, stent longitudinal integrity has got a lot of interest [3, 5]. The term LSD was used to describe some problems affecting it. Stent elongation was considered as one form of the LSD. Although the stent length in this study was longer than the known stent length, LSD was not considered because the incidence of LSD was generally uncommon, 1.1% [4, 18], and the incidence of stent elongation was rare too, 0.19% [3]. It was caused mainly by mechanical factors as occurring during retrieval of a protection wire [19].

In addition to M-L, three important factors were found to determine the stent length: the lesion length, the affected vessel, and the minimal vessel diameter. All these factors correlated directly with the difference in stent length except the minimal vessel diameter that had an inverse correlation. These factors may explain the difference in the stent length in nearly 60% of cases. Other factors such as balloon elongation may be present.

IVUS examination remains a crucial tool for studying the longitudinal stent architecture so that the researchers compare the stent length by using the recent investigative tools like optical coherence tomography with IVUS measurements [20]. Also, the stent length proved to affect the outcomes of percutaneous coronary intervention. It was associated with increased major adverse cardiac events (a composite of death, myocardial infarction, and target vessel revascularization). On the other hand, the use of IVUS during coronary stenting with long stents reduced the risk of these events significantly (hazard ratio, 0.47 and 0.57 for stent lengths measured from 23 to 32 mm and more than 32 mm, respectively) [21].

### Study limitations

Several stent types were included in this study. Third-generation DES was not included in the study. The number of used stents with 4.0 mm stent diameter was only four stents that rendered the comparison between the M-L and E-E length insignificant.

## Conclusion

To conclude, the length of BES is usually longer, and in addition to the manufacturer-stated length and foreshortening data, other factors such as lesion length, minimal diameter of lesion EEM, and vessel site determine the stent length. In our study, longitudinal stent foreshortening is not dominant, and the stent length is different from the manufacturer-stated length, so it should be put in mind on doing longitudinal stent measurements.

## Data Availability

The manuscript data is available on request from the corresponding author.

## Abbreviations

A-A length: Area-to-area stent length measured by IVUS
BES: Balloon-expandable stent
BMS: Bare metal stent
DES: Drug-eluting stent
E-E length: Edge-to-edge stent length measured by IVUS
EEM: External elastic membrane
IVUS: Intravascular ultrasound
LSD: Longitudinal stent deformation
M-L length: Manufacturer-stated length
OCT: Optical coherence tomography
